# Genome-Wide Identification and Expression Analysis of BAG Family in Sweet Potato and Its Two Diploid Relatives

**DOI:** 10.3390/ijms26189053

**Published:** 2025-09-17

**Authors:** Xiaochen Zhang, Qingchang Liu, Hong Zhai, Ning Zhao, Shaopei Gao, Huan Zhang, Shaozhen He

**Affiliations:** 1 Frontiers Science Center for Molecular Design Breeding, Key Laboratory of Crop Heterosis and Utilization (MOE)/Key Laboratory of Sweet Potato Biology and Biotechnology, Ministry of Agriculture and Rural Affairs/Beijing Key Laboratory of Crop Genetic Improvement/Laboratory of Crop Heterosis & Utilization and Joint Laboratory for International Cooperation in Crop Molecular Breeding, Ministry of Education, College of Agronomy & Biotechnology, China Agricultural University, Beijing 100193, China; xchenzhang@163.com (X.Z.); liuqc@cau.edu.cn (Q.L.); zhaihong@cau.edu.cn (H.Z.); zhaoning2012@cau.edu.cn (N.Z.); spgao@cau.edu.cn (S.G.); 2 Sanya Institute, China Agricultural University, Sanya 572025, China

**Keywords:** sweet potato, *BAG*, tuberous root development, tissue-specific expression, abiotic and biotic stress

## Abstract

The Bcl-2 associated athanogene (BAG) family is a multifunctional group of proteins that perform diverse functions, ranging from apoptosis to tumorigenesis. In plants, BAGs play a key role in growth, autophagy, and stress response. However, the BAG family has not been explored in sweet potato. In this study, we identified 15, 14, and 14 *BAGs* in cultivated hexaploid sweet potato (*I. batatas*, 2*n* = B_1_B_1_B_2_B_2_B_2_B_2_ = 6*x* = 90) and its two diploid relatives *I. trifida* (2*n* = 2*x* = 30) and *I. triloba* (2*n* = 2*x* = 30) by sequence alignment, genome structure analysis, and phylogenetic characterization. Based on their phylogenetic relationships with *Arabidopsis*, we divided these BAGs into three subfamilies. Protein physicochemical properties, chromosome localization, collinearity and Ka/Ks analysis, phylogenetic relationships, gene structures, promoter *cis*-elements, protein interaction networks, and expression patterns were systematically investigated to explore the possible functions of these 43 *BAGs* in the development and abiotic and biotic stress response of sweet potato. The results suggested that homologous *BAGs* have differentiated functions and play various vital roles in plant growth, tuberous root development, and abiotic and biotic stress response in sweet potato and its two diploid relatives. This work provides a comprehensive comparison and understanding of the *BAG* genes in sweet potato and its two diploid relatives, supplying a theoretical foundation for their functional study and further facilitating the molecular breeding of sweet potato.

## 1. Introduction

The BAG (Bcl-2 associated athanogene) family is a multifunctional group of proteins that perform diverse functions, ranging from apoptosis to tumorigenesis [1]. This evolutionarily conserved group of proteins is defined by the BAG domain, which enables interaction with and regulation of the Hsp70 (70 kDa heat shock protein) family of molecular chaperones. In particular, some BAGs contain extra domains such as the ubiquitin-like (UBL) domain or the calmodulin-binding IQ motif, which may be related to specific plant functions like hormone signaling and cell death regulation [2,3,4].

*BAG* genes have been identified in animals, yeasts, and plants and are believed to function as adapter proteins forming complexes with signaling molecules and molecular chaperones. As early as 1995, the first BAG family member, BAG-1, was identified in a screen of a mouse embryonic cDNA library using protein interaction cloning with human Bcl-2 as bait [5]. All identified human BAG proteins (BAG-1-6) each possess the characteristic BAG domain near the C-terminal end, except for BAG5, which contains four BAG domains [6]. BAG family proteins are widely involved in a variety of biological processes, such as apoptosis, tumor formation, stress response, neural differentiation, and the cell cycle [7]. For example, BAG-1L enhances androgen receptors transcriptional activity in an Hsp70-dependent manner, resulting in resistance to anti-androgen drug therapy [8].

In contrast to the detailed functional characterization of some mammalian BAG proteins, studies on the BAG family in plants remain relatively limited. In plants, the number of BAGs varies among different species, i.e., 7 in *Arabidopsis*, 6 in rice, and 21 in maize [5,9,10,11,12]. These genes are involved in several physiological processes, such as growth, development, and, in particular, abiotic and biotic stress responses. In *Arabidopsis*, AtBAG1 and AtBAG2 are essential for the normal growth of plants, and *AtBAG1*-overexpressing plants show a delay in growth [13,14]. AtBAG5, which contains an IQ motif, promotes leaf senescence under darkness, causing chlorophyll loss and ROS accumulation [15]. AtBAG2, AtBAG6 and AtBAG7 contribute to drought and heat stress responses, and the *bag2/bag6* double mutant exhibits enhanced survival under drought treatment compared to WT, while mutation in the *AtBAG6* gene can enhance the basic thermotolerance of *Arabidopsis* [16]. Moreover, AtBAG4 appears to protect plants from cold stress by inhibiting programmed cell death (PCD), and overexpression of *AtBAG4* increases salt tolerance in *Arabidopsis* and rice [5]. In addition, BAGs also play important roles in biotic stress. The *atbag6* knockout lines exhibit enhanced susceptibility to the necrotrophic fungus *Botrytis cinerea*, and the AtBAG6 -AtBAGP1-AtAPCB1 complexes participate together in antifungal defense [3,9]. In both *Arabidopsis* and soybean, AtBAG7 functions in the ER-nucleus signaling pathway and mediates defense responses against *Phytophthora capsici* [17]. In rice, OsBAG4 is necessary to trigger PCD and to improve rice tolerance to *Xanthomonas oryzae* pv. *oryzae* and *Magnaporthe oryzae* [18]. The OsEBR1-OsBAG4 module orchestrates innate immune homeostasis and coordinates the trade-off between defense and growth in plants [18]. Interestingly, AtBAG4, the *Arabidopsis* homolog of OsBAG4, enhances plant tolerance to abiotic stresses while exerting no discernible influence on disease resistance [19]. In wheat, when TaHsp70 and TaBAG2 are co-overexpressed in plants, the excessive TaBAG2 binds to excess TaHsp70 protein to release HSF, thereby increasing thermotolerance in plants [20]. However, the function of BAG proteins in sweet potato remains poorly understood.

Sweet potato (*Ipomoea batatas* (L.) Lam., 2*n* = B_1_B_1_B_2_B_2_B_2_B_2_ = 6*x* = 90), a member of the family Convolvulaceae, is a major root tuber crop cultivated globally for both industrial and bioenergy applications [21]. It provides abundant carbohydrates, dietary fiber, carotenoids, vitamins, and essential micronutrients, making it a key contributor to nutritional security. Owing to its remarkable adaptability, sweet potato also supports food security for smallholder farmers, particularly in Africa and Southeast Asia [22]. Despite its agricultural and nutritional value, sweet potato production is increasingly threatened by both abiotic and biotic stresses [23]. Among these, salinity and drought are two major abiotic constraints that can severely reduce agricultural output and threaten global food security. As for biotic stress, root rot causes a 30% loss in production, compromises quality, and occasionally results in complete crop failure [24,25]. In China, approximately 120,000 ha of the main sweet potato-producing regions are affected by root rot, leading to an annual economic loss exceeding 500 million yuan. Improving sweet potato yield and quality therefore requires a deeper understanding of the genetic mechanisms underlying root development and stress responses. However, such efforts have long been hindered by the high complexity of the sweet potato genome, which arises from its polyploidy.

Recent advances in genome sequencing, including the assemblies of the hexaploid cultivar Taizhong 6 and two closely related diploid species, *Ipomoea trifida* NCNSP0306 and *Ipomoea triloba* NCNSP0323, now provide high-quality genomic resources for systematic identification and characterization of key gene families [26,27].

In this study, we identified 43 *BAG* genes from *I. batatas* (15), *Ipomoea trifida* (14), and *Ipomoea triloba* (14) and classified them into three subgroups. We systematically investigated protein physicochemical properties, chromosome localization, collinearity analysis, phylogenetic relationships, gene structures, *cis*-elements of promoters, and protein interaction networks of *BAGs* in sweet potato. Furthermore, RNA-seq data were used to examine the evolutionary divergence, tissue-specific expression, and expression patterns of *BAG* genes between two sweet potato cultivars and their two diploid relatives. Comparative transcriptomic analysis revealed functional differences in development and responses to both abiotic and biotic stresses between the two sweet potato varieties.

## 2. Results

### 2.1. Identification and Characteristics of BAGs in Sweet Potato and Its Two Diploid Relatives

Cultivated hexaploid sweet potato differs markedly from its diploid relatives, which lack the capacity to form tuberous roots. To achieve a comprehensive catalog of BAG family members in sweet potato and its two diploid relatives, we identified candidate genes using homology-based searches with blastp and HMMER, followed by validation of conserved BAG domains using CD-search. A total of 43 *BAGs* were identified in *I. batatas* (15), *I. trifida* (14), and *I. triloba* (14), which were named after “*Ib*”, “*Itf*”, and “*Itb*”, respectively. These were designated as *IbBAGs*, *ItfBAGs*, and *ItbBAGs*, respectively, and systematically numbered based on their physical order along the chromosomes. The physicochemical properties were analyzed using the sequence of IbBAGs (Table 1). The genomic length of the 15 *IbBAGs* ranged from 1182 bp (*IbBAG12*) to 5977 bp (*IbBAG9*), with CDS length ranging from 531 bp (*IbBAG12*) to 4029 bp (IbBAG9). Correspondingly, protein sizes varied between 176 aa (IbBAG12) and 1342 aa (IbBAG9), with molecular weight (MW) ranging from 20.16 kDa (IbBAG12) to 149.71 kDa (IbBAG9). The isoelectric point (pI) of IbBAG9 (5.55), IbBAG12 (5.47), and IbBAG15 (4.57) were lower than 7.0 among all IbBAGs, indicating that they were acidic proteins. The pI of other IbBAGs distributes from 8.55 (IbBAG13) to 9.8 (IbBAG1), indicating that they were basic proteins. All IbBAGs possessed phosphorylation sites for serine, threonine, and tyrosine residues. The aliphatic index of all IbBAGs is less than 100. All IbBAGs exhibited negative grand averages of hydropathicity (GRAVY) consistent with an overall hydrophilic character. Predicted subcellular localization suggested distribution across the plastid, cytoplasm, or nucleus. To provide correspondence with the latest sweet potato reference genome Tanzania, the mapping of *IbBAGs* from the Taizhong6 genome to the six haplotypes of the Tanzania genome is presented in Table S1 [28].

**Figure 1:**
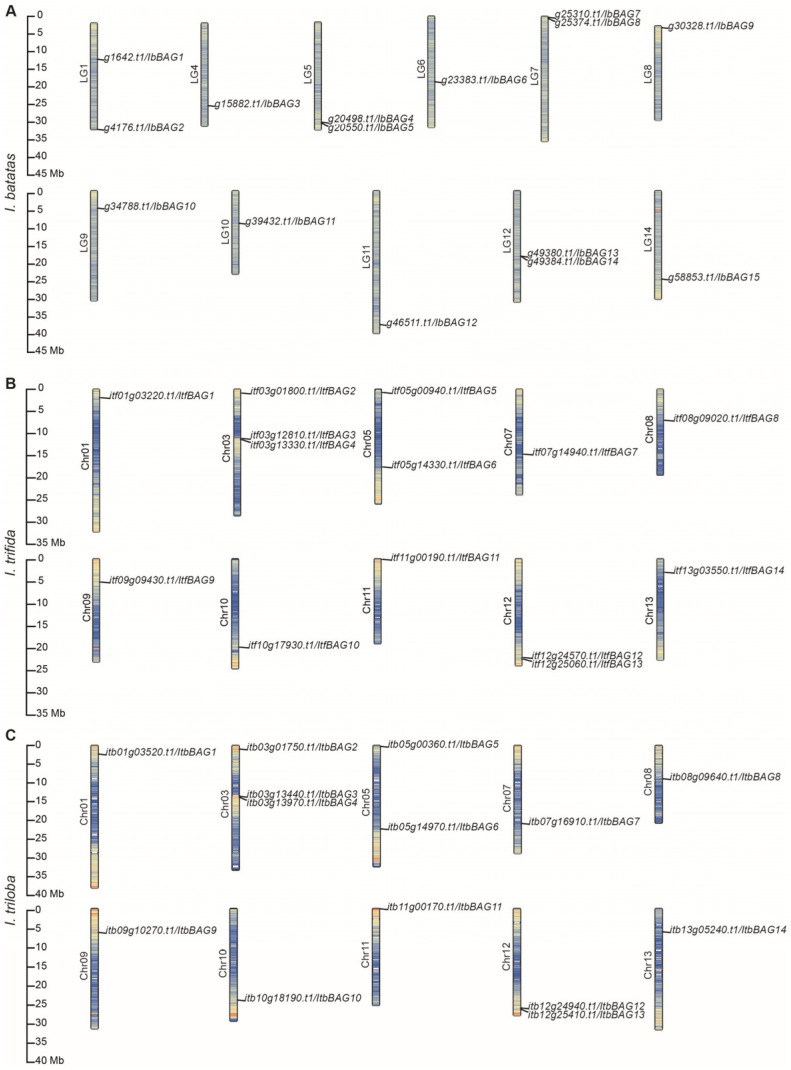
Chromosomal localization and distribution of *BAGs* in *I. batatas* (**A**), *I. trifida* (**B**), and *I. triloba* (**C**). The bars represent chromosomes, the chromosome numbers are displayed on the left side, and the gene names are displayed on the right side. Each gene location is exhibited on the line.

The *BAGs* were distributed 11 chromosomes of *I. batatas*, and 10 chromosomes of *I. trifida*, and *I. triloba* (Figure 1). In *I. batatas*, two *IbBAGs* were detected on LG1/5/7/12, one on LG4/6/8/9/10/11/14 (Figure 1A). In *I. trifida* and *I. triloba*, the distribution of *BAGs* on Chr01/7/8/9/10/11/13 (1), Chr03 (3), Chr5/12 (2) was similar (Figure 1B,C). These results indicate that evolutionary variation and expansion of the *BAG* gene family have shaped differences in chromosomal distribution and gene copy number between sweet potato and its diploid relatives.

### 2.2. Collinearity and Ka/Ks Analysis of BAG Genes

**Figure 2:**
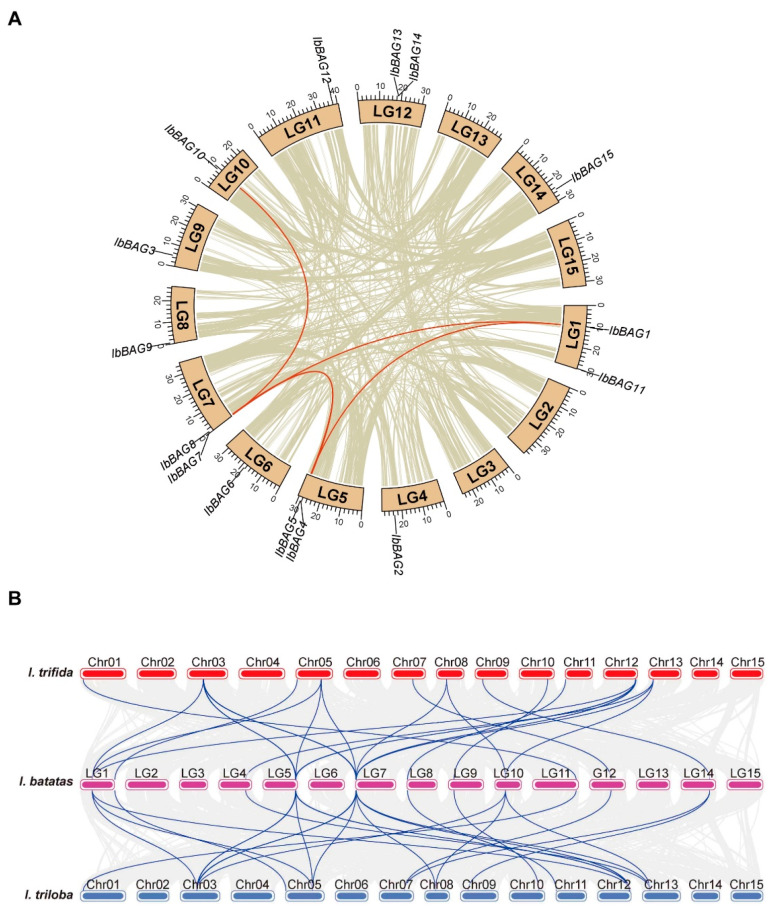
Gene location and collinearity analysis of the *BAG* genes in *I. batatas*. (**A**) The genes were located on different chromosomes. Duplicated gene pairs are linked with a deep red line. (**B**) Collinearity analysis of the *BAG* genes. Pink, red, and blue blocks denote chromosomes of *I. batatas*, *I. trifida*, and *I. triloba*, respectively. Dark blue curves represent the syntenic relationships among the three species.

Gene duplication, including dispersed (DSD) and tandem duplication (TD), is a major driver of gene family expansion [29]. To identify gene duplication events, we detected intraspecific collinear gene pairs among the *BAG* gene family in sweet potato via TBtools [30]. The results revealed that a total of five segmental duplication pairs were found in *I. batatas*, including *IbBAG1*/*IbBAG7*, *IbBAG1*/*IbBAG5*, *IbBAG4*/*IbBAG8*, *IbBAG5*/*IbBAG7*, and *IbBAG7*/*IbBAG11* (Figure 2A). These findings suggested that dispersed duplication events played a pivotal role in the expansion of the *IbBAG* gene family (Figure 2A).

Additionally, the synteny among sweet potato and its two diploid relatives was investigated. Most *IbBAGs* possessed one to three orthologous genes with *ItfBAGs* and *ItbBAGs*, except for *IbBAG6* and *IbBAG14* which had no orthologous genes, suggesting functional conservation during domestication (Figure 2B). These orthologous gene pairs were located on different chromosomes, indicating that the duplication in the *BAG* gene family contributed to the process of evolution from diploid to hexaploid.

To evaluate the evolutionary pressures on *IbBAGs*, the nonsynonymous (Ka) and synonymous (Ks) substitution rates were calculated for five homologous gene pairs. As shown in Table 2, the Ka values were substantially lower than the Ks values across all pairs, resulting in Ka/Ks ratios of <1. These results implied that these *BAGs* were subjected to strong purifying selection pressures over the evolutionary process.

### 2.3. Phylogenetic Relationship of BAGs in Sweet Potato and Its Two Diploid Relatives

**Figure 3:**
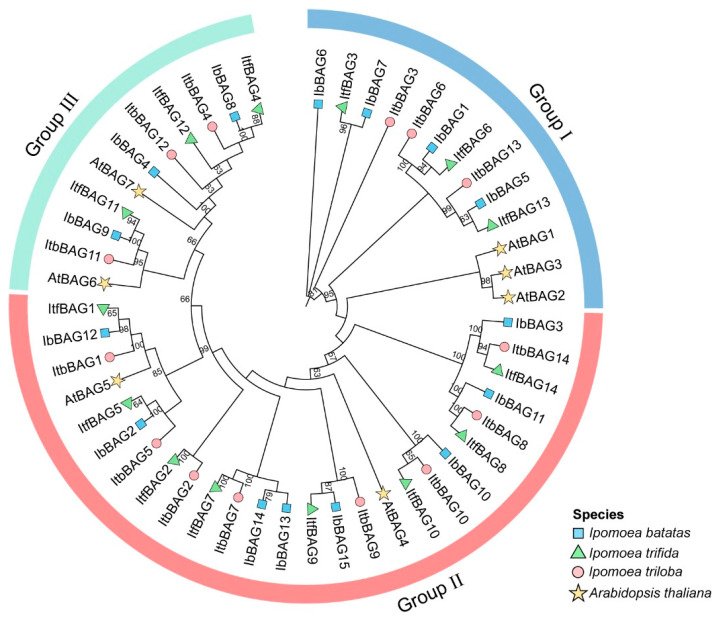
Phylogenetic analysis of the BAGs in *I. batatas*, *I. trifida*, *I. triloba*, and *Arabidopsis*. A total of 50 BAGs were divided into three subgroups (Group I to III) according to the evolutionary distance. The blue squares represent the 15 IbBAGs in *I. batatas*. The green triangles represent the 14 ItfBAGs in *I. trifida*. The red circles represent the 14 ItbBAGs in *I. triloba*; the yellow stars represent the 7 AtBAGs in *Arabidopsis*.

To study the evolutionary relationship of BAGs in *I. batatas*, *I. trifida*, *I. triloba*, and *Arabidopsis*, we constructed a phylogenetic tree for 50 BAGs of these four species (i.e., *I. batatas* (15), *I. trifida* (14), *I. triloba* (14), and *Arabidopsis* (7)) (Figure 3). The BAGs were distributed across the tree and clustered into three groups (Group I–III) based on evolutionary distance (Figure 3). The specific distribution of BAGs was as follows (total: *I.batatas*, *I. trifida*, *I. triloba*, *Arabidopsis*): Group I (13:4,3,3,3), Group II (26:8,8,8,2), and Group III (11:3,3,3,2) (Figure 3; Table S2). This phylogenetic topology suggests both conserved evolutionary patterns and lineage-specific diversification events.

Furthermore, we further performed a broader phylogenetic analysis using 77 BAG proteins from 6 plant species (i.e., *I. batatas* (15), *I. trifida* (14), *I. triloba* (14), *Arabidopsis* (7), rice (6), and maize (21)). They were divided into three subgroups (Group I to III) (Figure S1), which indicated that the evolutionary relationship of BAGs was relatively conserved in plants.

### 2.4. Conserved Motif and Exon–Intron Structure Analysis of BAGs in Sweet Potato and Its Two Diploid Relatives

**Figure 4:**
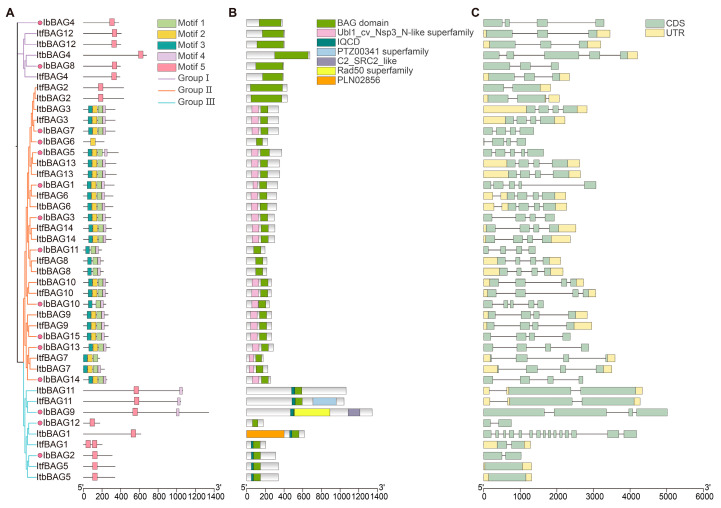
Conserved motifs and exon-intro structure analysis of BAG family in *I. batatas*, *I. trifida*, and, *I. triloba*. (**A**) The phylogenetic tree showed that BAGs were distributed to three subgroups in the left, and the five conserved motifs were shown in different colors. The red circle represents the IbBAGs. (**B**) Conserved domain structures of BAGs. The box represents different domain. (**C**) Exon–intron structures of *BAGs*. The green boxes, yellow boxes, and black lines represent the exons, introns, and UTRs, respectively.

Furthermore, we analyzed sequence motifs in the 15 IbBAGs, 14 ItfBAGs, and 14 ItbBAGs using the MEME, and identified the five most conserved motifs. (Figure 4A and Figure S2). The motif distribution varied among the three BAG groups. In Group I, all BAG proteins contained only motif 5 (Figure 4A). Similarly, the majority of proteins in Group III also contained only motif 5, with the exception of IbBAG9, ItfBAG11, and ItbBAG11, which additionally possessed motif 4 (Figure 4A). In contrast, most BAGs in Group II contained three to four motifs (motif 1–4), except for ItfBAG2 (motif 5), ItbBAG2 (motif 5), and IbBAG6 (motif 2) (Figure 4A).

BAG proteins are characterized by a common conserved region located near the C terminus, termed the BAG domain that mediates direct interaction with the ATPase domain of Hsp70/Hsc70 molecular chaperones [31]. All BAG proteins contain a BAG domain (Figure 4B). Moreover, BAG proteins usually contain additional domains. For example, the ubiquitin-like (UBL) domain, which is necessary for stress tolerance and involved in a proteasome-mediated protein degradation, is present in most BAGs of Group II, with the exception of the BAGs that did not contain motif 2 (Figure 4B) [2]. IQ (isoleucine-glutamine) motif containing D (IQCD) is unique to plants [9]. In Group III, most of the BAGs contain an IQCD near the BAG domain, except for IbBAG12, suggesting that they may be involved in unique biological processes in plants (Figure 4B) [2,32]. Moreover, some BAG proteins contain unique domains. ItfBAG11 specifically contained a PTZ00341 domain (Ring-infected erythrocyte surface antigen), IbBAG9 uniquely contained a C2_SRC2_like domain (involved in Ca^2+^ dependent protein binding) and a Rad50 domain (associated with recombination, recombinational repair, and non-homologous end joining), and ItbBAG1 contained a PLNO2856 domain (related to fumarylacetoacetase activity) (Figure 4B).

To investigate structural diversity among *BAGs*, we analyzed the exon–intron organization (Figure 4C). All the *BAGs* contained one to fifteen exons. In detail, *BAGs* of Group I contained three to five exons, *BAGs* of Group II contained two to five exons, and most *BAGs* of Group III contained one to four exons. Notably, *ItbBAG1* contained fifteen exons (Figure 4C). The exon–intron structures of some homologous *BAGs* were different in *I. batatas* compared with those in *I. trifida* and *I. triloba*, such as *IbBAG10* (which contained 5 exons), *ItfBAG10* (which contained 4 exons), and *ItbBAG10* (which contained 4 exons) in Group II, *IbBAG2* (which contained 2 exons), and *ItfBAG5* and *ItbBAG5* (which contained 1 exon) in Group III (Figure 4C). These results indicate that the *BAG* family may have undergone a lineage-specific differentiation event in the sweet potato genome.

### 2.5. Cis-Element Analysis in the Promoter of IbBAGs in Sweet Potato

**Figure 5:**
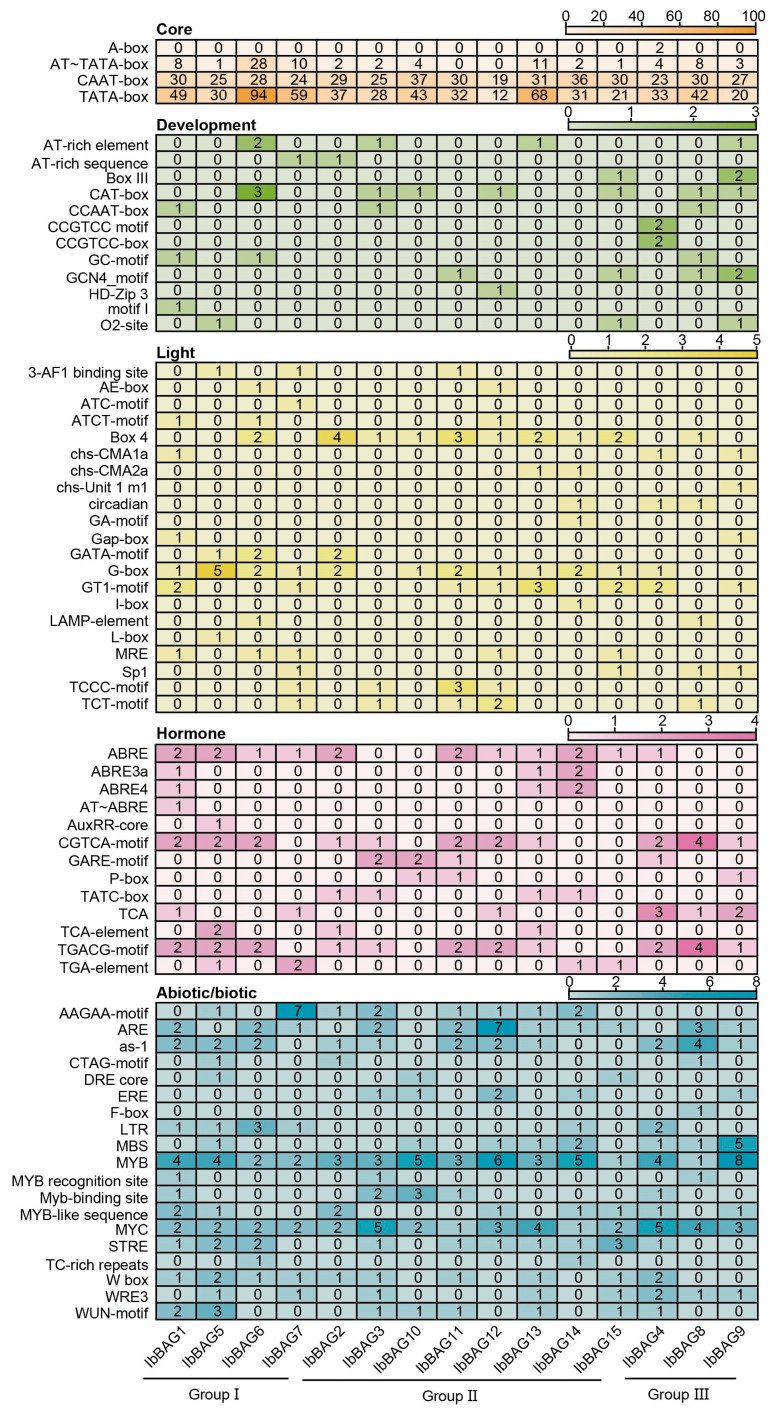
*Cis*-elements analysis of *IbBAG*s in *I. batatas*. The *cis*-elements were divided into five categories. The degree of different colors represents the number of *cis*-elements in the *IbBAGs* promoters.

Promoter *cis*-elements play key roles in regulating gene functions related to plant development, hormone signaling, and stress responses. To explore the regulatory potential of *IbBAGs*, we analyzed the 1500 bp upstream promoter regions of all 15 *IbBAGs*. Based on predicted functions, the identified elements were classified into five categories: core elements, development-related, light-responsive, hormone-responsive, and abiotic/biotic stress-responsive elements (Figure 5). All *IbBAG* promoters contained core elements, such as CAAT-box and TATA-box (Figure 5). Most genes also contained development-related elements, including the AT-rich element (a binding site for AT-rich DNA binding proteins; found in *IbBAG3*, -*6*, -*9*, and -*13*), CAT-box (associated with meristem expression; found in *IbBAG3*, -*6*, -*8*, -*9*, -*10*, -*12*, and -*15*), and GCN4 (involved in seed-specific expression; found in *IbBAG8*, -*9*, -*11*, and -*15*) (Figure 5). Furthermore, light-responsive elements were abundant in *IbBAG* promoters, such as BOX4, G-box, and MRE (Figure 5). Hormone-responsive elements were present in all IbBAGs, including ABRE (ABA-responsive), TATC-box (GA-responsive), CGTCA and TGACG-motifs (MeJA-responsive), TCA (SA-responsive), and TGA-element (IAA-responsive) (Figure 5).

Notably, a large number of abiotic and biotic stress-responsive elements were identified across the 15 *IbBAGs*. All *IbBAGs* contained the MYB element, which is involved in responses to drought, salt stress, and pathogen resistance [33,34,35]. Similarly, all *IbBAGs* also contained the MYC element, a key regulator in the ABA and JA signaling pathways that mediate responses to cold and wounding [36,37]. In addition, STRE was also identified in several *IbBAG* promoters (except for *IbBAG2*, -*7*, -*8*, -*9*, and -*10*), which participates in abiotic stress response such as heat shock, osmotic stress, and oxidative stress. Most *IbBAGs* (except for *IbBAG8*, -*9*, -*10*, -*12*, and -*14*) contained W-box elements, which mediate defense responses against fungal pathogens [38,39]. These results indicate that *IbBAGs* likely participate in regulating plant growth and development, hormone signaling crosstalk, and adaptation to abiotic and biotic stresses in sweet potato.

### 2.6. Protein Interaction Network of IbBAGs in Sweet Potato

**Figure 6:**
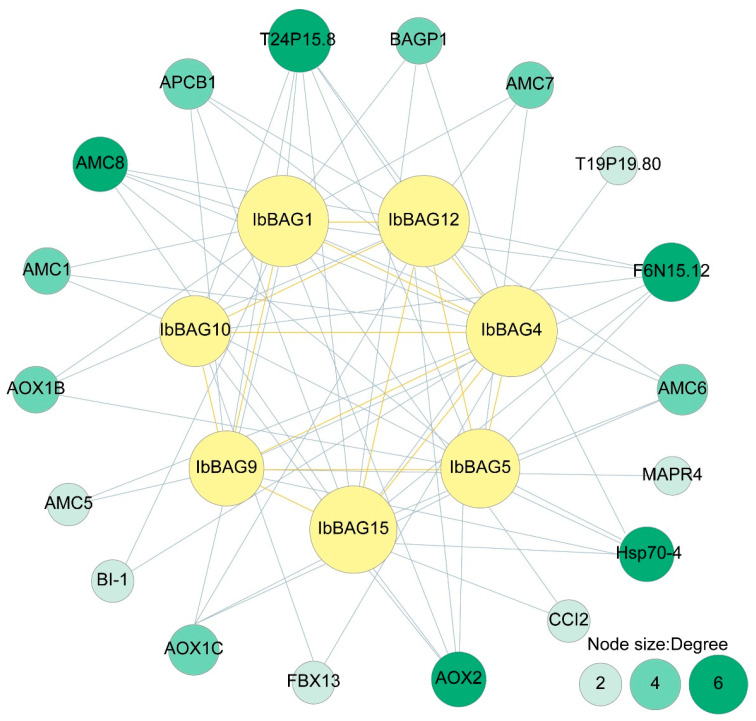
Functional interaction networks of IbBAGs in *I. batatas* according to orthologues in *Arabidopsis*. Network nodes represent proteins, and lines represent protein-protein associations. The interactions between different BAGs are represented by yellow lines. The interactions between BAGs and other proteins are represented by green lines. The node size and different colors represent the number of proteins which interact with each other.

To explore the potential regulatory network of IbBAGs, we constructed a protein–protein interaction network based on *Arabidopsis* orthologs (Figure 6). The analysis predicted that several IbBAGs (IbBAG1, -4, -5, -9, -10, -12 and -15) could interact with other BAG family members. In addition, BAGs were predicted to interact with a range of functional proteins, such as metacaspases (AMC1, AMC5, AMC6, AMC7, and AMC8) which are regulators of cell death; aspartyl protease APCB1, a key component in plant basal immunity [40]. Notably, IbBAG1 and IbBAG4 also interacted with Bax inhibitor 1 (BI-1), which is a suppressor of apoptosis [41]. These results show that BAGs might play a key role in hypersensitive response (HR)-associated PCD responding to fungal infection. BAGs also interacted with ubiquinol oxidase (AOX1B, AOX1C and AOX2), which are involved in the mitochondrial electron transport chain [42]. IbBAG4 and IbBAG10 could interact with F-box protein FBX13, which responds to environmental stress. Since STRING data are based on *Arabidopsis* orthologs, these results should be interpreted cautiously and require experimental validation. Collectively, these results suggest that IbBAGs are associated with stress responses and modulation of cell death in sweet potato.

### 2.7. Expression Analysis of BAGs in Sweet Potato and Its Two Diploid Relatives

#### 2.7.1. Expression Analysis in Various Tissues

**Figure 7:**
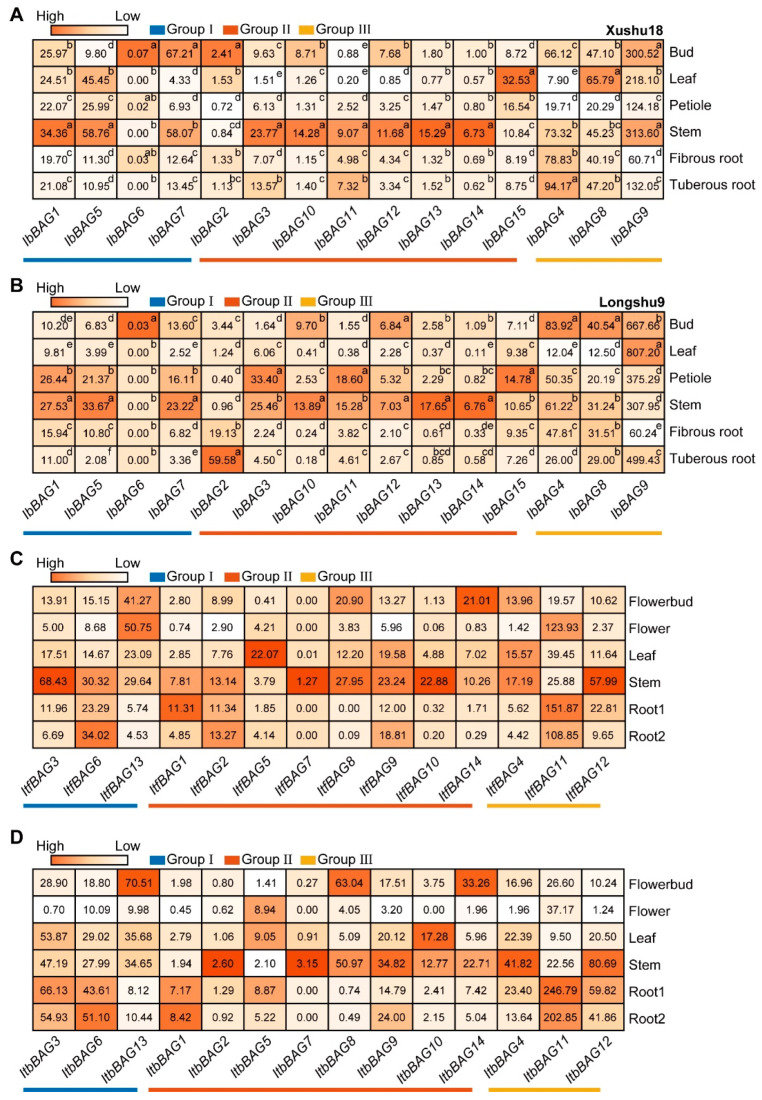
Gene expression patterns of *BAGs* in different tissues of *I. batatas*, *I. trifida*, and *I. triloba*. (**A**,**B**) Expression analysis as determined by RNA-seq in bud, leaf, petiole, stem, fibrous root, and tuberous root of *I. batata* in Xushu18 (**A**) and Longshu9 (**B**). The FPKM value is shown in the boxes. Different lowercase letters indicate a significant difference in each *IbBAG* at *p* < 0.05 based on one-way ANOVA. (**C**,**D**) Gene expression patterns of *ItfBAGs* (**C**) and *ItbBAGs* (**D**) in flower bud, flower, leaf, stem, root 1, and root 2 of *I. trifida* (**C**) and *I. triloba* (**D**) as determined by RNA-seq. FPKM was shown in the boxes.

To investigate the potential biological function of *IbBAGs* in growth and development, the expression levels in six representative tissues of two sweet potato varieties (i.e., bud, leaf, petiole, stem, fibrous root, and tuberous root) of *I. batatas*, *I. trifida*, and *I. triloba* were analyzed using RNA-seq data (Figure 7).

In Xushu18 (late-bulking, root rot-resistant) and Longshu9 (early-bulking, root rot-susceptible), most *IbBAGs* in Group I and Group II showed higher expression levels in the stem in both varieties. Notably, some BAGs exhibited distinct expression patterns between the two varieties. In Group I, *IbBAG6* was lowly expressed (≤0.07) in all tissues. In Group II, *IbBAG2* was highly expressed in bud of Xushu18, but highly expressed in tuberous root of Longshu9. In Group III, *IbBAG4* was highly expressed in tuberous roots of Xushu18 but highly expressed in bud of Longshu9. This differential expression pattern suggests that *BAG* genes may play roles in tissue-specific regulatory mechanisms.

In the two diploid relatives *I. trifida* and *I. triloba*, most *BAGs* in Group II also highly expressed in stems, except for *BAG1*, *BAG5*, *BAG11* and *BAG14*. In Group I, *ItfBAG3* was highly expressed in stem, whereas *ItbBAG3* was highly expressed in root. In Group III, *BAGs* displayed similar expression pattern: *Itf*/*ItbBAG4* and *Itf*/*ItbBAG12* exhibited high expression levels in stem, while *Itf*/*ItbBAG11* showed high expression levels in root. These results indicate that *BAGs* exhibit different expression patterns and play important roles in the stress responses and development of sweet potato and the two diploids.

#### 2.7.2. Expression Analysis in Different Development Stages

**Figure 8:**
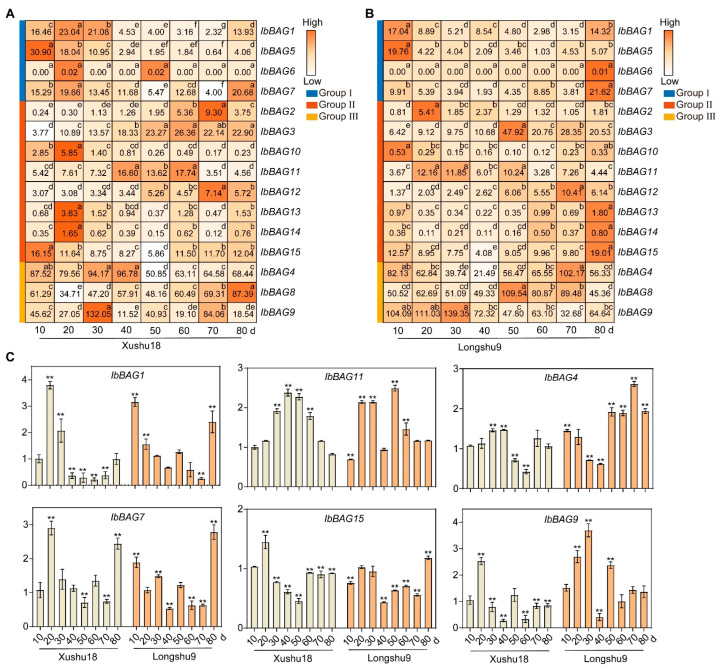
Gene expression patterns of *IbBAGs* in different root development stages (i.e., 10 d, 20 d, 30 d, 40 d, 50 d, 60 d, 70 d, and 80 d) as determined by RNA-seq in Xushu18 (**A**) and Longshu9 (**B**). The FPKM value is shown in the boxes. Different lowercase letters indicate a significant difference at *p* < 0.05 based on one-way ANOVA. (**C**) Expression of *IbBAGs* in different root development stages (i.e., 10 d, 20 d, 30 d, 40 d, 50 d, 60 d, 70 d, and 80 d). The error bars indicate ±SD (*n* = 3). The expression of 10 d of Xushu18 was considered as “1”. Asterisks indicate a significant difference at *p* < 0.05 based on Student’s *t*-test.

We further evaluated the expression levels of *IbBAGs* by RNA-seq in different development stages of sweet potato roots (i.e., 10 d, 20 d, 30 d, 40 d, 50 d, 60 d, 70 d, and 80 d) (Figure 8). Interestingly, most *BAGs* exhibited opposite temporal expression patterns between Xushu18 and Longshu9. In Xushu18, most *IbBAGs* were highly expressed at early root developmental stages, except for *IbBAG2*, -*3*, -*8*, -*11*, and -*12*. However, most *IbBAGs* showed high expression levels at later stages in Longshu9, except *IbBAG1*, -*2*, -*5*, -*9*, -*10*, and -*11*. Notably, *IbBAG9* showed a higher expression trend in Xushu18, which may respond to the initiation of tuberous formation. In Longshu9, *IbBAG11* and -*9* exhibited similar expression patterns. The qRT-PCR results were consistent with the transcriptome data (Figure 8C). These results suggest that *IbBAGs* may be involved in different root development stages in Xushu18 and Longshu9.

#### 2.7.3. Expression Analysis Under Abiotic Stress

**Figure 9:**
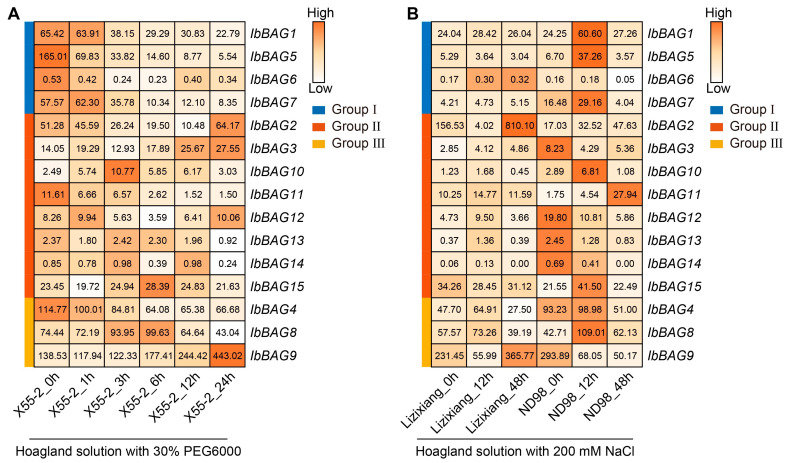
Gene expression patterns of *IbBAGs* under drought and salt stresses as determined by RNA-seq. (**A**) Expression analysis of *IbBAGs* under PEG treatment in a drought-tolerant variety, i.e., Xu55-2. (**B**) Expression analysis of *IbBAGs* under NaCl treatment in a salt-sensitive variety, i.e., Lizixiang, and a salt-tolerant line, i.e., ND98. The FPKM value is shown in the boxes.

To explore the possible roles of *IbBAGs* in abiotic stress responses, we analyzed the expression patterns of *IbBAGs* using the RNA-seq data of a drought-tolerant variety (Xu55-2) under drought stress, and the RNA-seq data of a salt-sensitive variety (Lizixiang) and a salt-tolerant line (ND98) under salt stress. Most *IbBAGs* in Groups II and III were induced by PEG treatment in Xu55-2, but all *IbBAGs* in Group I and *IbBAG2*, -*4*, -*11*, -*12* were down-regulated. Moreover, *IbBAG1*, -*4*, -*5*, -*7*, -*8*, -*10*, -*11*, and -*15* were induced by NaCl treatment in ND98. Furthermore, *IbBAG8*, -*10* and -*15* were induced by both PEG and NaCl treatments in Xu55-2 and ND98 (Figure 9).

In addition, we also analyzed the expression patterns of *BAGs* using the RNA-seq data of *I. trifida* and *I. triloba* under drought and salt treatments. *ItfBAG5* and -*11* and *ItbBAG3*, -*5*, -*7*, -*9* and -*11* were induced by both drought and salt treatments (Figure S3A,B). Taken together, these results indicate that BAGs are differentially expressed in response to various abiotic stresses in sweet potato and its two diploid relatives.

We further analyzed the expression patterns of *BAGs* using the RNA-seq data of *I. trifida* and *I. triloba* under cold and heat treatments (Figure S3C–F). Overall, all *ItfBAGs* and most *ItbBAGs* were down-regulated by both cold and heat stresses. Notably, *ItbBAG1*, -*5*, -*6*, -*9*, and -*11* were induced by cold stress, whereas *ItfBAG2*, -*5*, -*11*, -*14* and *ItbBAG4*, -*5*, -*11* exhibited increased expression under heat stress.

#### 2.7.4. Expression Analysis in Different Varieties in a Root Rot Field

**Figure 10:**
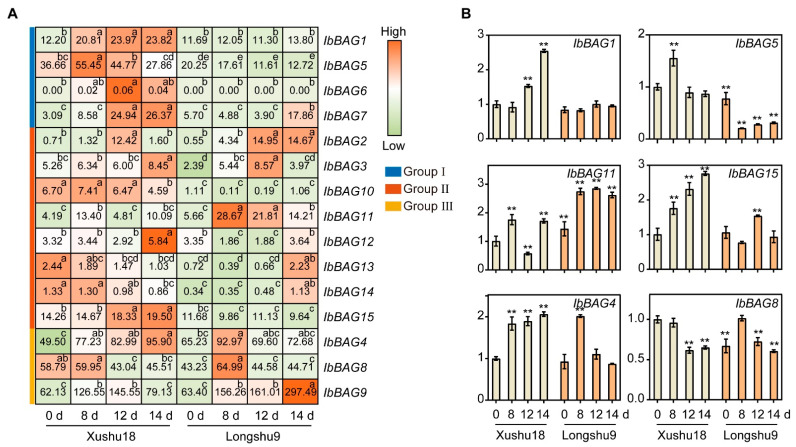
Gene expression patterns of *IbBAGs* in Xushu18 and Longshu9 in a severe root rot field 0, 8, 12, 14 days after planting (DAP) by RNA-seq and qRT-PCR. (**A**) The FPKM value is shown in the boxes. Different lowercase letters indicate a significant difference for each *IbBAG* at *p* < 0.05 based on one-way ANOVA. (**B**) Expression of *IbBAGs* on different days with root rot infection. The error bars indicate ±SD (*n* = 3). The expression of 10 d of Xushu18 was considered as “1”. Asterisks indicate a significant difference at *p* < 0.05 based on Student’s *t*-test.

Since *IbBAGs* are involved in the biotic stress responses of sweet potato, and root rot is a major disease that significantly reduces global sweet potato yield and quality, we further analyzed the expression levels of *IbBAGs* in two sweet potato cultivars Xushu18 (root rot-resistant) and Longshu9 (root rot-susceptible) in a severe root rot field after planting (DAP) (Figure 10) [24,25,43]. Interestingly, the expression levels of most *IbBAGs* in Xushu18 tended to be higher than those in Longshu9. The results showed that most *BAGs* were rapidly induced in Xushu18 after 8 days of root rot infection, except for *IbBAG8*, -*10*, -*13*, and -*14*. However, in Longshu9, *IbBAG5* and *IbBAG10* showed a decreasing trend. Notably, in response to root rot infection, expression levels of *IbBAG1*, -*4*, -*7*, and -*15* exhibited a gradual increase over the 14-day experimental period in Xushu18. Moreover, *IbBAG9* and *IbBAG11* showed higher expression levels in Longshu9 than in Xushu18. The qRT-PCR results were consistent with the transcriptome data (Figure 10B). These results indicated that *IbBAGs* may be involved in root rot resistance responses of sweet potato roots.

## 3. Discussion

The BAG protein family plays diverse roles, including mediating apoptosis and tumorigenesis in animals, and is also involved in pathogen defense, abiotic and biotic stress responses, and development in plants [5,16,44]. However, their roles and regulatory mechanisms in sweet potato remain poorly understood. In this study, we systematically identified *BAG* genes and compared their characteristics between the cultivated hexaploid sweet potato and its two diploid relatives. This comprehensive genomic analysis provides a foundation for elucidating BAG functions and supports future molecular breeding efforts in sweet potato.

### 3.1. Evolution of the BAGs Gene Family in Sweet Potato and Its Two Diploid Relatives

In this study, a total of 43 *BAGs* were identified from sweet potato and its two diploid relatives. The number of *BAGs* identified in *I. batatas* (15) is greater than *I. trifida* (14) and *I. triloba* (14) (Figure 1; Table S2). Compared with other model plants, the *BAG* gene family in sweet potato is moderately expanded, with more members than *Arabidopsis* (7) and rice (6), but fewer than maize (21) (Figure S1) [5,11,12]. These findings also suggest that the *BAG* gene family in sweet potato has undergone lineage-specific gene gains during its evolutionary history. Consistent with this, genomic synteny analysis revealed substantial chromosomal differentiation among the three species [45]. The chromosome localization and distribution of *BAGs* were different between *I. batatas*, *I. trifida*, and *I. triloba*, such as 11 chromosomes contained *BAG* genes in *I. batatas*, but 10 in *I. trifida* and *I. triloba* (Figure 1). All *BAG* genes have distinct subcellular localizations (plastid, cytoplasmic, and nucleus), which could lead to functional differences (Table 1).

Gene duplication is a major driver of gene family expansion and functional innovation. Among the 15 *IbBAG* genes, we identified five segmentally duplicated gene pairs, all with Ka/Ks < 1 (Figure 2A, Table 2), suggesting that purifying selection has maintained their structural integrity while allowing for possible functionalization. Such duplication events may provide sweet potato with additional *BAG* gene copies that can be differentially regulated under abiotic or biotic stress, contributing to its superior stress tolerance compared with its diploid relatives. Notably, for gene pairs with Ks values exceeding 1–2, synonymous sites may be approaching saturation, which could reduce the accuracy of Ka/Ks estimation. Therefore, the Ka/Ks ratios of these gene pairs should be interpreted with caution.

Phylogenetic analysis divided the 43 *BAG* genes into three distinct groups (Group I–III), with *IbBAG6* forming an outgroup, indicating early evolutionary divergence and potentially unique biological functions. Notably, *IbBAGs* and *ItfBAGs* clustered more closely compared with *ItbBAGs*, suggesting a closer evolutionary relationship between sweet potato and *I. trifida* (Figure 3). Notably, sweet potato *BAG* genes form an evolutionarily distinct monophyletic clade, demonstrating lineage-specific divergence from other plants (Figure S1). This lineage-specific divergence may reflect adaptive evolution of *BAG* genes in response to the complex environmental conditions encountered by sweet potato during domestication and cultivation.

Motif composition analysis revealed five highly conserved motifs among BAG proteins, including the canonical BAG domain, which was conserved across sweet potato and its two diploid relatives (Figure 4A,B). The BAG proteins within the same phylogenetic group generally shared similar motif architectures, suggesting functional conservation. Notably, in Group II, almost all BAGs contain the Ubi domain and motif 1–4; however, IbBAG6, ItfBAG2, and ItbBAG2 show different motif patterns, containing only the BAG domain (Figure 4A). Therefore, BAGs with similar motifs have similar biological functions during plant life in sweet potato.

While intronic transcription imposes metabolic and temporal constraints on plants, these noncoding elements critically enable proteome diversification through exon shuffling and alternative splicing. Introns further orchestrate enhanced gene expression, nuclear mRNA export, and transcription-coupled regulation [46,47,48]. Here, exon–intron organization varied substantially among orthologous *BAG* genes in the three species (Figure 4C). For example, *IbBAG10* contained five exons, whereas its orthologs *ItfBAG10* and *ItbBAG10* contained only four; *IbBAG2* had two exons compared to a single exon in *ItfBAG5* and *ItbBAG5*; and *IbBAG4* had four exons, whereas *ItfBAG12* and *ItbBAG12* contained three. Such variation in exon–intron structure can facilitate alternative splicing and transcriptional flexibility, potentially contributing to the functional diversification of *BAG* genes in sweet potato and its relatives. This structural divergence likely enhances the ability of sweet potato BAGs to respond dynamically to developmental cues and environmental stresses, highlighting their potential importance in molecular breeding for stress resilience.

Gene expression in polyploids exhibits considerable complexity. Notably, parenchyma tissues in some plant species display characteristic polyploid features [49]. Furthermore, the number of plastids per cell and the corresponding compartments for starch deposition may also vary with ploidy [50]. Although such phenomena have not yet been reported in sweet potato, these possibilities should be considered in future research. Despite the comparable sizes of the BAG gene families between hexaploid sweet potato (*I. batatas*, 15 members) and its diploid relatives (*I. trifida* and *I. triloba*, 14 members each), differences in allele dosage are likely to result from their distinct ploidy levels, suggesting that ploidy may play a decisive role in this process. Consistently, allele dosage has been shown to significantly contribute to phenotypic variation in the roots of sweet potato, indicating that ploidy level is a critical determinant for root organ formation [43].

### 3.2. Different Expression Pattern of BAGs on Root Development Stage in Two Sweet Potato Varieties

In plants, accumulated studies have indeed shown that BAGs play important and diverse roles in many fields, including plant growth and development [9,11,44]. In *Arabidopsis*, AtBAG1 and AtBAG2 are critical for normal plant growth, with *AtBAG1* overexpression causing a delay in growth [13,14]. AtBAG5, which carries an IQ motif, induces leaf senescence under dark conditions, leading to chlorophyll degradation and ROS accumulation [15].

In this study, most *IbBAGs* exhibited opposite temporal expression patterns between the late-bulking, root rot-resistant variety Xushu18 and the early-bulking, root rot-susceptible variety Longshu9 (Figure 8). For example, *IbBAG15*, which contained GCN4 motif, was highly expressed at 10 d in Xushu18 but at 80 d in Longshu9. Additionally, all Group III *IbBAGs* showed consistently high expression across all developmental stages. Moreover, *IbBAG9* which contained the most motif involved in development, showed the highest expression levels throughout root development. These observations suggest that the temporal regulation of *IbBAGs* is cultivar-specific and may underlie the differential growth dynamics and stress resilience observed between Xushu18 and Longshu9. Moreover, the early activation of specific *IbBAGs* in Xushu18 may contribute to enhanced root development and preemptive defense against root rot, while delayed expression in Longshu9 could partly explain its susceptibility.

Overall, our results highlight that precise temporal and cultivar-dependent expression of *IbBAGs* is likely a key factor in coordinating root growth with stress responses in sweet potato.

### 3.3. Different Functions of BAGs on Abiotic Stress Response Between Two Sweet Potato Varieties and Its Two Diploid Relatives

The ability to respond to abiotic stresses is crucial for plant survival under fluctuating environmental conditions. BAG proteins play diverse roles in mediating plant responses to various abiotic stresses, including salinity, drought, and extreme temperatures. In *Arabidopsis*, AtBAG4 protects plants from cold stress by inhibiting PCD, and overexpression of *AtBAG4* increases salt tolerance in both *Arabidopsis* and rice. In rice, overexpression of *OsBAG4* enhanced the tolerance to salt stress in the transgenic plants [51]. In wheat, co-overexpression of *TaHsp70* and *TaBAG2* leads to an accumulation of excess TaBAG2, which binds to surplus TaHsp70 to release HSF and enhances thermotolerance in plants [20].

In this study, *IbBAG* genes exhibited differential expression patterns in response to PEG treatment in Xu55-2 and NaCl treatment in ND98. Specifically, seven *IbBAGs* were upregulated under PEG-induced drought stress in Xu55-2, while eight *IbBAGs* were induced under salt stress in ND98. Notably, *IbBAG5* was sharply repressed in Xu55-2 by PEG treatment; however, *IbBAG5* was induced by NaCl treatment in ND98 (Figure 9). Moreover, *IbBAG3* and *IbBAG10* were induced by both PEG and NaCl treatment. These findings suggest potential functional divergence among members of the *IbBAG* gene family, implying that distinct IbBAGs may play specialized regulatory roles in sweet potato adaptation to drought and salt stress.

Furthermore, the diploid relatives *I. trifida* and *I. triloba* provide valuable genetic resources for identifying functional genes that may have been lost during sweet potato domestication. In the two diploid relatives, *ItfBAG5* and -*11* and *ItbBAG3*, -*5*, -*7*, -*9* and -*11* were induced by both drought and salt treatments (Figure S3A,B).

In addition, cold and heat stresses predominantly suppress the expression of *BAG* genes, whereas a subset of members, particularly *ItbBAG11* and *ItfBAG11*, exhibit strong induction under extreme temperatures (Figure S3C–F). It is established that BAG proteins interact with HSP/HSC70 chaperone complexes and play a regulatory role in PCD. The up-regulation of these specific genes suggests their potential involvement in maintaining protein homeostasis and protecting cells from thermal damage.

These *BAG* genes represent promising candidates for future breeding programs aimed at improving abiotic stress tolerance in cultivated sweet potato.

### 3.4. Different Functions of BAGs on Biotic Stress Response Between Two Sweet Potato Varieties and Its Two Diploid Relatives

Plants are frequently exposed to biotic stresses such as microbial pathogens and insect attacks. BAG proteins have been reported to participate in plant responses to biotic stresses. In *Arabidopsis*, the *atbag6* knockout lines show increased susceptibility to *Botrytis cinerea*, with AtBAG6, AtBAGP1, and AtAPCB1 forming complexes that contribute to antifungal defense [4]. In rice, OsBAG4 plays a crucial role in triggering PCD and enhancing rice tolerance to *Xanthomonas oryzae* pv. *oryzae* and *Magnaporthe oryzae* [18]. The OsEBR1-OsBAG4 module regulates immune homeostasis and balances defense with growth in plants [18].

In this study, *IbBAGs* were differentially expressed in response to root rot between two sweet potato varieties Xushu18 (root rot-resistant) and Longshu9 (root rot-susceptible) (Figure 10). Notably, most *IbBAG* genes exhibited higher expression levels in Xushu18 than in Longshu9 during root rot infection, including *IbBAG1*, -*4*, -*5*, -*6*, -*7*, -*10*, -*13*, -*14*, and -*15*. In particular, *IbBAG1*, -*3*, -*4*, -*7*, and -*15* were induced in the whole period of root rot infection, suggesting their potential key roles in root rot resistance. Additionally, several genes, such as *IbBAG5*, -*8*, -*10*, -*13*, and -*14*, displayed a sharp and rapid response to infection in Xushu18, indicating their possible involvement in early-stage defense. In particular, *IbBAG5* and *IbBAG12*, which were strongly induced by root rot, exhibited significantly higher expression levels in Xushu18 than in Longshu9, further supporting their roles in resistance. Collectively, these findings suggest that *IbBAGs* participate in early defense responses against root rot, likely by contributing to cytoprotection under stress conditions and inhibiting plant PCD [9]. Overall, *IbBAGs* may act as critical regulators of root rot resistance, although their precise regulatory mechanisms in biotic stress responses warrant further investigation.

## 4. Materials and Methods

### 4.1. Identification of BAGs

The genome sequences of *I. batatas*, *I. trifida*, and *I. triloba* were retrieved from the *Ipomoea* Genome Hub (https://ipomoea-genome.org/, accessed on 30 July 2025) and Sweetpotato Genomics Resource (http://sweetpotato.plantbiology.msu.edu/, accessed on 30 July 2025). The corresponding genome annotation files were also downloaded, and the predicted protein sequences derived from these annotations were used as the reference datasets for *BAG* gene identification. To reliably identify all members of the *BAG* gene family, we employed a combination of three complementary strategies: the BLAST algorithm was used to identify predicted *BAGs* using all *AtBAGs* from the *Arabidopsis* genome database (https://www.arabidopsis.org/, accessed on 30 July 2025) as queries by BLAST+ v2.16.0 (BLASTP, E-value ≤ 1 × 10^−5^, low-complexity filtering enabled “-seg yes”), the Pfam database was used to extract BAG domain (PF02179), and HMMER 3.0 software was employed to identify potential *BAG*s (hmmsearch, E-value ≤ 1 × 10^−5^, domain coverage ≥ 50%). Finally, all candidate BAG proteins were confirmed using SMART (http://smart.embl-heidelberg.de/, accessed on 30 July 2025) and CD-search (https://www.ncbi.nlm.nih.gov/Structure/cdd/wrpsb.cgi, accessed on 30 July 2025).

### 4.2. Chromosomal Distribution of BAGs

Chromosomal location data were downloaded from the *Ipomoea* Genome Hub (https://ipomoea-genome.org/, accessed on 30 July 2025) and Sweetpotato Genomics Resource (http://sweetpotato.plantbiology.msu.edu/, accessed on 30 July 2025). The identified *BAG* genes were mapped to the chromosomes using TBtools software (v2.310, South China Agricultural University, Guangzhou, China) [30].

### 4.3. Collinearity and Ka/Ks Ratios of BAG Genes

Segmental duplications of the *IbBAG* gene family were analyzed using MCScanX (E-value cutoff of 1 × 10^−10^) in TBtools v2.336 [30]. Segmental duplication relationships were visualized with the Advanced Circos function. The same approach was applied to examine collinearity between sweet potato and its diploid relatives, *I. trifida* and *I. triloba*. For Ka/Ks analysis, the Ka and Ks values of collinear *IbBAG* gene pairs were calculated using the “Simple Ka/Ks Calculator” module in TBtools. The coding sequences (CDS) of each gene pair were first extracted and aligned using codon-based alignment within TBtools. The Nei-Gojobori (NG) model was used to estimate Ka and Ks values, and gaps or ambiguous codons were automatically trimmed during the calculation.

### 4.4. Phylogenetic Analysis of BAGs

Multiple sequence alignment of the deduced amino acid sequences of the BAGs from I. batatas, I. trifida, I. triloba, Arabidopsis, Zea mays, and Oryza sativa were aligned with Clustal X, and the alignment was imported into MEGA11 to create a phylogenetic tree using the neighbor-joining method with 1000 bootstrap replicates. Then, the phylogenetic tree was constructed using iTOL (http://itol.embl.de/, accessed on 2 September 2025).

### 4.5. Domain Identification and Conserved Motifs Analysis of BAGs

The conserved motifs of BAGs were analyzed by MEME (https://meme-suite.org/meme/, accessed on 31 July 2025), and the maximum number was set to 5. The remaining motifs parameters were set to their default values.

### 4.6. Exon–Intron Structures and Promoter Analysis of BAGs

The exon–intron structures of *BAGs* were analyzed using GSDS 2.0 (http://gsds.gao-lab.org/, accessed on 31 July 2025) and visualized with TBtools v2.336. The *cis*-elements in the approximately 1500 bp promoter region of *BAGs* were predicted by PlantCARE (http://bioinformatics.psb.ugent.be/webtools/plantcare/html/, accessed on 10 August 2025).

### 4.7. Protein Interaction Network of BAGs

Protein interaction networks of BAGs were predicted using STRING (https://cn.string-db.org/, accessed on 10 August 2025) based on *Arabidopsis* homologous proteins (orthologs identified via reciprocal BLASTP, E-value ≤ 1 × 10^−5^, sequence identity ≥ 50%; medium confidence score ≥ 0.4), and visualized with Cytoscape v3.10.2 [52].

### 4.8. Transcriptome Analysis

The RNA-seq data of *ItfBAGs* and *ItbBAGs* were downloaded from the Sweetpotato Genomics Resource (http://sweetpotato.plantbiology.msu.edu/, accessed on 10 August 2025). The RNA-seq data of *IbBAGs* in *I. batatas* were obtained from related research in our laboratory [53,54]. The expression levels of BAGs were calculated as fragments per kilobase of exon per million fragments mapped (FPKM), and the heat maps were constructed by TBtools v2.336 [30].

### 4.9. qRT-PCR Analysis of BAGs

Total RNA was extracted from the roots of the two varieties (Xushu18 and Longshu9) grown in the root rot field at multiple time points (10, 20, 30, 40, 50, 60, 70, and 80 days after planting, DAP). Three biological replicates were used, each with five plants. The transcript levels were measured using qRT-PCR, with the sweet potato *β-Actin* gene (GenBank AY905538) as an internal control. Gene expression was quantified using the comparative *C*_T_ method [43].

## 5. Conclusions

In this study, we conducted a comprehensive analysis of the *BAG* gene family in cultivated hexaploid sweet potato (*I. batatas*, 2*n* = B_1_B_1_B_2_B_2_B_2_B_2_ = 6*x* = 90) and its two diploid relatives, *I. trifida* (2*n* = 2*x* = 30) and *I. triloba* (2*n* = 2*x* = 30). Based on genome and transcriptome resources, we identified 15, 14, and 14 *BAG* genes in these species, respectively. Multiple aspects of their genomic features were systematically investigated, including protein physicochemical properties, chromosome localization, collinearity and Ka/Ks analysis, phylogenetic relationships, gene structures, promoter *cis*-elements, and predicted protein interaction networks. Expression profiles were further examined using RNA-seq and qRT-PCR data, focusing on tissue specificity, tuberous root development, and responses to both abiotic and biotic stresses.

The results indicated that there was a differentiation in the functions of homologous *BAGs*, and each *BAG* gene played different vital roles in the growth and development of sweet potato and its two diploid relatives, as well as in abiotic and biotic stress response,. Notably, *IbBAGs* displayed cultivar-specific expression patterns during root development and diverse responses to abiotic and biotic stresses, with key members likely coordinating growth, enhancing root rot resistance, and maintaining cellular homeostasis. These findings provide a solid foundation for future functional studies and the development of stress-resilient sweet potato varieties.

## Figures and Tables

**Table 1:** Characterization of *IbBAGs* in sweet potato.

Gene ID	Gene Name	pI	Molecular Weight/kDa	Genomic Length/bp	CDS Length/bp	Phosphorylation Site			Protein Size/aa	Aliphatic Index	GRAVY	Subcellular Locations
						Ser	Thr	Tyr				
g1642	*IbBAG1*	9.8	36.31	3757	993	13	8	7	330	85.33	−0.353	Plastid
g4176	*IbBAG2*	9.3	35.47	1377	927	11	9	4	308	76.2	−0.783	Cytoplasmic
g15882	*IbBAG3*	9.73	32.62	2263	885	9	7	7	294	83.88	−0.438	Cytoplasmic
g20498	*IbBAG4*	9.03	43.21	3555	1140	8	3	6	379	85.99	−0.539	Plastid
g20550	*IbBAG5*	9.42	41.26	2435	1125	16	9	3	374	77.38	−0.651	Nucleus
g23383	*IbBAG6*	9.3	24.91	1395	669	4	3	2	222	88.6	−0.314	Plastid
g25310	*IbBAG7*	9.43	37.42	2994	1020	10	10	6	339	73.3	−0.634	Nucleus
g25374	*IbBAG8*	9.37	45.82	2401	1191	7	8	8	396	77.1	−0.686	Plastid
g30328	*IbBAG9*	5.55	149.72	5977	4029	49	37	22	1342	63.92	−0.814	Nucleus
g34788	*IbBAG10*	8.86	26.58	2143	726	6	5	1	241	78.13	−0.502	Plastid
g39432	*IbBAG11*	9.49	21.96	1656	588	2	4	2	195	78	−0.579	Plastid
g46511	*IbBAG12*	5.47	20.16	1182	531	9	3	1	176	75.28	−0.517	Plastid
g49380	*IbBAG13*	8.55	31.61	3107	858	15	7	2	285	81.37	−0.412	Nucleus
g49384	*IbBAG14*	9.01	28.01	3057	762	14	5	2	253	85.89	−0.399	Plastid
g58853	*IbBAG15*	4.57	29.03	3191	801	9	7	3	266	81.95	−0.489	Nucleus

CDS, coding sequence; MW, molecular weight; pI, isoelectric point; Ser, serine; Thr, threonine; Tyr, tyrosine.

**Table 2:** Ka/Ks analysis results.

Seq_1	Seq_2	Ka	Ks	Ka/Ks	Effective Length	Average S-Sites	Average N-Sites	Type of Selection
*IbBAG1*	*IbBAG7*	0.25	1.54	0.16	768	180.33	587.67	Purify selection
*IbBAG1*	*IbBAG5*	0.33	1.34	0.25	819	189	630	Purify selection
*IbBAG5*	*IbBAG7*	0.21	1.36	0.15	981	222.33	758.67	Purify selection
*IbBAG4*	*IbBAG8*	0.16	0.94	0.17	1080	247.42	832.58	Purify selection
*IbBAG7*	*IbBAG11*	0.41	2.67	0.15	555	120.08	434.92	Purify selection

## Data Availability

All data needed to evaluate the conclusions in the paper are present in the paper and/or the Supporting Information. The RNA-seq data of *I. trifida* and *I. triloba* can be accessed in the Sweetpotato Genomics Resource (http://sweetpotato.plantbiology.msu.edu/, accessed on 10 August 2025). The other RNA-seq data are available on request from the corresponding author.

## Supplementary Materials

The following supporting information can be downloaded at: https://www.mdpi.com/article/10.3390/ijms26189053/s1.

## References

[1] Antoku K., Maser R.S., Scully W.J., Delach S.M., Johnson D.E. (2001). Isolation of Bcl-2 Binding Proteins That Exhibit Homology with BAG-1 and Suppressor of Death Domains Protein. Biochem. Biophys. Res. Commun..

[2] Takayama S., Reed J.C. (2001). Molecular Chaperone Targeting and Regulation by BAG Family Proteins. Nat. Cell Biol..

[3] Doong H., Vrailas A., Kohn E.C. (2002). What’s in the ‘BAG’?—A Functional Domain Analysis of the BAG-Family Proteins. Cancer Lett..

[4] Li Y., Dickman M. (2016). Processing of AtBAG6 Triggers Autophagy and Fungal Resistance. Plant Signal. Behav..

[5] Doukhanina E.V., Chen S., van der Zalm E., Godzik A., Reed J., Dickman M.B. (2006). Identification and Functional Characterization of the BAG Protein Family in *Arabidopsis thaliana*. J. Biol. Chem..

[6] Kang C.H., Jung W.Y., Kang Y.H., Kim J.Y., Kim D.G., Jeong J.C., Baek D.W., Jin J.B., Lee J.Y., Kim M.O. (2006). AtBAG6, a Novel Calmodulin-Binding Protein, Induces Programmed Cell Death in Yeast and Plants. Cell Death Differ..

[7] Behl C. (2016). Breaking BAG: The Co-Chaperone BAG3 in Health and Disease. Trends Pharmacol. Sci..

[8] Froesch B.A., Takayama S., Reed J.C. (1998). BAG-1L Protein Enhances Androgen Receptor Function. J. Biol. Chem..

[9] Kabbage M., Dickman M.B. (2008). The BAG Proteins: A Ubiquitous Family of Chaperone Regulators. Cell. Mol. Life Sci..

[10] Nawkar G.M., Maibam P., Park J.H., Woo S.G., Kim C.Y., Lee S.Y., Kang C.H. (2017). In Silico Study on *Arabidopsis BAG* Gene Expression in Response to Environmental Stresses. Protoplasma.

[11] Zhou H., Li J., Liu X., Wei X., He Z., Hu L., Wang J., Duan M., Xie G., Wang J. (2021). The Divergent Roles of the Rice Bcl-2 Associated Athanogene (BAG) Genes in Plant Development and Environmental Responses. Plants.

[12] Farid B., Saddique M.A.B., Tahir M.H.N., Ikram R.M., Ali Z., Akbar W. (2025). Expression Divergence of BAG Gene Family in Maize under Heat Stress. BMC Plant Biol..

[13] Fang S., Li L., Cui B., Men S., Shen Y., Yang X. (2013). Structural Insight into Plant Programmed Cell Death Mediated by BAG Proteins in *Arabidopsis thaliana*. Acta Crystallogr. Sect. D Biol. Crystallogr..

[14] Lee D.W., Kim S.J., Oh Y.J., Choi B., Lee J., Hwang I. (2016). *Arabidopsis* BAG1 Functions as a Cofactor in Hsc70-Mediated Proteasomal Degradation of Unimported Plastid Proteins. Mol. Plant.

[15] Cui B., Fang S., Xing Y., Shen Y., Yang X. (2015). Crystallographic analysis of the *Arabidopsis thaliana* BAG5-calmodulin protein complex. Acta Crystallogr. Sect. F Struct. Biol. Commun..

[16] Arif M., Men S., Nawaz A.F., Li X., Xu L., Yang X., Fahad S., Ahmad P., Xu R., Li L. (2024). Bcl-2-Associated Athanogene (BAG) Co-Chaperones: Key Players in Multiple Abiotic and Biotic Stress Tolerance in Plants. J. Plant Growth Regul..

[17] Zhou Y., Yang K., Cheng M., Cheng Y., Li Y., Ai G., Bai T., Xu R., Duan W., Peng H. (2021). Double-Faced Role of Bcl-2-Associated Athanogene 7 in Plant–*Phytophthora* Interaction. J. Exp. Bot..

[18] You Q., Zhai K., Yang D., Yang W., Wu J., Liu J., Pan W., Wang J., Zhu X., Jian Y. (2016). An E3 Ubiquitin Ligase-BAG Protein Module Controls Plant Innate Immunity and Broad-Spectrum Disease Resistance. Cell Host Microbe.

[19] Hoang T.M.L., Moghaddam L., Williams B., Khanna H., Dale J., Mundree S.G. (2015). Development of Salinity Tolerance in Rice by Constitutive-Overexpression of Genes Involved in the Regulation of Programmed Cell Death. Front. Plant Sci..

[20] Ge S., Kang Z., Li Y., Zhang F., Shen Y., Ge R., Huang Z. (2016). Cloning and Function Analysis of BAG Family Genes in Wheat. Funct. Plant Biol..

[21] Liu Q.C. (2017). Improvement for Agronomically Important Traits by Gene Engineering in *Sweetpotato*. Breed. Sci..

[22] El Sheikha A.F., Ray R.C. (2017). Potential Impacts of Bioprocessing of Sweet Potato: Review. Crit. Rev. Food Sci. Nutr..

[23] Bartels D., Sunkar R. (2005). Drought and Salt Tolerance in Plants. Crit. Rev. Plant Sci..

[24] Scruggs A.C., Quesada-Ocampo L.M. (2016). Etiology and Epidemiological Conditions Promoting *Fusarium* Root Rot in *Sweetpotato*. Phytopathology.

[25] Kim S., Kim T.H., Chung M.-N., Lee Y., Lee I.B., Lee H., Park W. (2022). Incidence Rates of Root Rot in *Sweetpotato* Caused by Cultivation Soil and Soil Microorganisms During Storage Periods. Front. Plant Sci..

[26] Wu S., Lau K.H., Cao Q.H., Hamilton J.P., Sun H.H., Zhou C.X., Eserman L., Gemenet D.C., Olukolu B.A., Wang H.Y. (2018). Genome Sequences of Two Diploid Wild Relatives of Cultivated *Sweetpotato* Reveal Targets for Genetic Improvement. Nat. Commun..

[27] Yang J., Moeinzadeh M.H., Kuhl H., Helmuth J., Xiao P., Haas S., Liu G.L., Zheng J.L., Sun Z., Fan W.J. (2017). Haplotype-resolved Sweet Potato Genome Traces Back Its Hexaploidization History. Nat. Plants.

[28] Wu S., Sun H., Zhao X., Hamilton J.P., Mollinari M., Gesteira G.D.S., Kitavi M., Yan M., Wang H., Yang J. (2025). Phased Chromosome-Level Assembly Provides Insight into the Genome Architecture of Hexaploid *Sweetpotato*. Nat. Plants.

[29] Chen H., Zhang Y., Feng S. (2023). Whole-Genome and Dispersed Duplication, Including Transposed Duplication, Jointly Advance the Evolution of *TLP* Genes in Seven Representative *Poaceae* Lineages. BMC Genom..

[30] Chen C., Wu Y., Li J., Wang X., Zeng Z., Xu J., Liu Y., Feng J., Chen H., He Y. (2023). TBtools-II: A “One for All, All for One” Bioinformatics Platform for Biological Big-Data Mining. Mol. Plant.

[31] Brive L., Takayama S., Briknarová K., Homma S., Ishida S.K., Reed J.C., Ely K.R. (2001). The Carboxyl-Terminal Lobe of Hsc70 ATPase Domain Is Sufficient for Binding to BAG1. Biochem. Biophys. Res. Commun..

[32] Yan J., He C., Zhang H. (2003). The BAG-Family Proteins in *Arabidopsis thaliana*. Plant Sci..

[33] Seo P.J., Xiang F., Qiao M., Park J.-Y., Lee Y.N., Kim S.-G., Lee Y.-H., Park W.J., Park C.-M. (2009). The MYB96 Transcription Factor Mediates Abscisic Acid Signaling during Drought Stress Response in *Arabidopsis*. Plant Physiol..

[34] Yang A., Dai X., Zhang W.-H. (2012). A R2R3-Type MYB Gene, *OsMYB2*, Is Involved in Salt, Cold, and Dehydration Tolerance in Rice. J. Exp. Bot..

[35] Zhang P., Wang R., Yang X., Ju Q., Li W., Lü S., Tran L.P., Xu J. (2020). The R2R3-MYB Transcription Factor AtMYB49 Modulates Salt Tolerance in *Arabidopsis* by Modulating the Cuticle Formation and Antioxidant Defence. Plant Cell Environ..

[36] Abe H., Urao T., Ito T., Seki M., Shinozaki K., Yamaguchi-Shinozaki K. (2003). *Arabidopsis* AtMYC2 (bHLH) and AtMYB2 (MYB) Function as Transcriptional Activators in Abscisic Acid Signaling. Plant Cell.

[37] Uji Y., Taniguchi S., Tamaoki D., Shishido H., Akimitsu K., Gomi K. (2016). Overexpression of *OsMYC2* Results in the Up-Regulation of Early JA-Rresponsive Genes and Bacterial Blight Resistance in Rice. Plant Cell Physiol..

[38] Eulgem T., Somssich I.E. (2007). Networks of WRKY Transcription Factors in Defense Signaling. Curr. Opin. Plant Biol..

[39] Rushton P.J., Somssich I.E., Ringler P., Shen Q.J. (2010). WRKY Transcription Factors. Trends Plant Sci..

[40] Li Y., Kabbage M., Liu W., Dickman M.B. (2016). Aspartyl Protease-Mediated Cleavage of BAG6 Is Necessary for Autophagy and Fungal Resistance in Plants. Plant Cell.

[41] Ishikawa T., Watanabe N., Nagano M., Kawai-Yamada M., Lam E. (2011). Bax Inhibitor-1: A Highly Conserved Endoplasmic Reticulum-Resident Cell Death Suppressor. Cell Death Differ..

[42] Albury M.S., Elliott C., Moore A.L. (2010). Ubiquinol-Binding Site in the Alternative Oxidase: Mutagenesis Reveals Features Important for Substrate Binding and Inhibition. Biochim. Biophys. Acta (BBA)—Bioenerg..

[43] Zhang H., Dai Z., Zhang X., Shang M., Gao X., Ma R., Zhao L., Zhang X., Liu Q., Zhai H. (2025). Natural Allelic Variations in *IbCHYR1*–*IbZnFR* Complex Regulate *Fusarium* Root Rot Resistance in Sweet Potato. Adv. Sci..

[44] Jiang H., Liu X., Xiao P., Wang Y., Xie Q., Wu X., Ding H. (2023). Functional Insights of Plant Bcl-2-Associated Ahanogene (BAG) Proteins: Multi-Taskers in Diverse Cellular Signal Transduction Pathways. Front. Plant Sci..

[45] Wan R., Liu J., Yang Z., Zhu P., Cao Q., Xu T. (2020). Genome-Wide Identification, Characterisation and Expression Profile Analysis of DEAD-Box Family Genes in Sweet Potato Wild Ancestor *Ipomoea trifida* under Abiotic Stresses. Genes Genom..

[46] Holland S., Blake C. (1987). Proteins, Exons and Molecular Evolution. Biosystems.

[47] Morello L., Gianì S., Troina F., Breviario D. (2011). Testing the IMEter on Rice Introns and Other Aspects of Intron-Mediated Enhancement of Gene Expression. J. Exp. Bot..

[48] Mukherjee D., Saha D., Acharya D., Mukherjee A., Chakraborty S., Ghosh T.C. (2018). The Role of Introns in the Conservation of the Metabolic Genes of *Arabidopsis thaliana*. Genomics.

[49] Cheniclet C., Rong W.Y., Causse M., Frangne N., Bolling L., Carde J.-P., Renaudin J.-P. (2005). Cell Expansion and Endoreduplication Show a Large Genetic Variability in Pericarp and Contribute Strongly to *Tomato* Fruit Growth. Plant Physiol..

[50] Butterfass T., Heilbrunn L.V., Beermann W., Rudkin G. (1979). Patterns of chloroplast reproduction. A developmental approach to protoplasmic plant anatomy. Cell Biology Monographs. Continuation of Protoplasmatologie.

[51] Wang J., Nan N., Li N., Liu Y., Wang T.-J., Hwang I., Liu B., Xu Z.-Y. (2020). A DNA Methylation Reader–Chaperone Regulator–Transcription Factor Complex Activates *OsHKT1;5* Expression during Salinity Stress. Plant Cell.

[52] Kohl M., Wiese S., Warscheid B. (2011). Cytoscape: Software for Visualization and Analysis of Biological Networks. Methods Mol. Biol..

[53] Zhang H., Zhang Q., Zhai H., Li Y., Wang X., Liu Q., He S. (2017). Transcript Profile Analysis Reveals Important Roles of Jasmonic Acid Signalling Pathway in the Response of *Sweet Potato* to Salt Stress. Sci. Rep..

[54] Zhu H., Zhou Y., Zhai H., He S., Zhao N., Liu Q. (2019). Transcriptome Profiling Reveals Insights into the Molecular Mechanism of Drought Tolerance in *Sweetpotato*. J. Integr. Agric..

