# Supplementary material for: Genome-Wide Identification and Expression Analysis of BAG Family in Sweet Potato and Its Two Diploid Relatives
Source: Int J Mol Sci. 2025 Sep 17;26(18):9053. doi: 10.3390/ijms26189053 (PMC12470027; doi:10.3390/ijms26189053)
Supplement: Supplementary file 1 [file ijms-26-09053-s001.zip › ijms-3841768-supplementary.pdf]

Table S1. *IbBAGs* homologous genes in Tanzania genome.

Gene	Tanzania Hap-A	Tanzania Hap-B	Tanzania Hap-C	Tanzania Hap-D	Tanzania Hap-E	Tanzania Hap-F
IbBAG1	*Ibat.Tzn_v2.05AG 015480.1*	*Ibat.Tzn_v2.05CG 015580.1*	*Ibat.Tzn_v2.05DG 015700.1*		*Ibat.Tzn_v2.05FG 014950.1*	*Ibat.Tzn_v2.05AG 015480.1*
IbBAG2	*Ibat.Tzn_v2.05AG 029870.1*	*Ibat.Tzn_v2.05BG 000990.1*	*Ibat.Tzn_v2.05CG 000740.1*	*Ibat.Tzn_v2.05DG 001450.1*		*Ibat.Tzn_v2.05EG 000670.1*
IbBAG3	*Ibat.Tzn_v2.13AG 004740.1*	*Ibat.Tzn_v2.13CG 004830.1*	*Ibat.Tzn_v2.13D G004570.1*	*Ibat.Tzn_v2.13EG 005310.1*	*Ibat.Tzn_v2.13FG 004680.1*	*Ibat.Tzn_v2.13AG 004740.1*
IbBAG4	*Ibat.Tzn_v2.12AG 022340.1*	*Ibat.Tzn_v2.12CG 026600.1*	*Ibat.Tzn_v2.12DG 024550.1*	*Ibat.Tzn_v2.12EG 028340.1*	*Ibat.Tzn_v2.12FG 024760.1*	*Ibat.Tzn_v2.12AG 022340.1*
IbBAG5	*Ibat.Tzn_v2.12AG 023470.1*	*Ibat.Tzn_v2.12BG 025080.1*	*Ibat.Tzn_v2.12CG 027020.1*		*Ibat.Tzn_v2.12EG 028760.1*	*Ibat.Tzn_v2.12FG 025260.1*
IbBAG6	*Ibat.Tzn_v2.01AG 006180.1*	*Ibat.Tzn_v2.10BG 006020.1*	*Ibat.Tzn_v2.06CG 021770.1*	*Ibat.Tzn_v2.15DG 014340.1*		*Ibat.Tzn_v2.15FG 015460.1*
IbBAG7		*Ibat.Tzn_v2.03BG 027040.1*		*Ibat.Tzn_v2.03EG 030000.1*	*Ibat.Tzn_v2.03FG 035200.1*	
IbBAG8	*Ibat.Tzn_v2.03AG 030970.1*	*Ibat.Tzn_v2.03BG 026520.1*	*Ibat.Tzn_v2.03DG 028400.1*	*Ibat.Tzn_v2.03EG 029610.1*	*Ibat.Tzn_v2.03FG 034260.1*	*Ibat.Tzn_v2.03AG 030970.1*
IbBAG9	*Ibat.Tzn_v2.11AG 000870.1*	*Ibat.Tzn_v2.11BG 000280.1*	*Ibat.Tzn_v2.11CG 000720.1*		*Ibat.Tzn_v2.11EG 000360.1*	
IbBAG10	*Ibat.Tzn_v2.10AG 019300.1*	*Ibat.Tzn_v2.10CG 017990.1*	*Ibat.Tzn_v2.10DG 024450.1*	*Ibat.Tzn_v2.10EG 019630.1*	*Ibat.Tzn_v2.10FG 020210.1*	*Ibat.Tzn_v2.10AG 019300.1*
IbBAG11		*Ibat.Tzn_v2.08BG 008200.1*	*Ibat.Tzn_v2.08CG 010360.1*	*Ibat.Tzn_v2.08DG 010550.1*	*Ibat.Tzn_v2.08EG 010380.1*	*Ibat.Tzn_v2.08FG 011200.1*
IbBAG12	*Ibat.Tzn_v2.01AG 004900.1*	*Ibat.Tzn_v2.01BG 006030.1*		*Ibat.Tzn_v2.01EG 002170.1*	*Ibat.Tzn_v2.01FG 006150.1*	*Ibat.Tzn_v2.01AG 004900.1*
IbBAG13	*Ibat.Tzn_v2.07AG 015660.2*	*Ibat.Tzn_v2.07BG 012420.1*	*Ibat.Tzn_v2.07CG 013870.1*	*Ibat.Tzn_v2.07DG 013280.1*	*Ibat.Tzn_v2.07EG 013900.2*	
IbBAG14	*Ibat.Tzn_v2.07AG 015660.2*	*Ibat.Tzn_v2.07BG 012420.1*	*Ibat.Tzn_v2.07CG 013870.1*	*Ibat.Tzn_v2.07DG 013280.1*	*Ibat.Tzn_v2.07EG 013900.2*	
IbBAG15	*Ibat.Tzn_v2.09AG 011320.1*	*Ibat.Tzn_v2.09BG 008260.1*	*Ibat.Tzn_v2.09CG 006910.2*	*Ibat.Tzn_v2.09DG 009730.1*	*Ibat.Tzn_v2.09EG 011030.3*	*Ibat.Tzn_v2.09FG 009600.3*

Table S2. Identification of *BAG* family genes in *I. batatas*, *I. trifida*, and *I. triloba*.

Arabidopsis	Homologous gene in *I. batatas*/ *I. trifida*/ *I. triloba*	Gene ID	Gene name
Group Ⅰ: *AT5G52060/AtBAG1* *AT5G62100/AtBAG2* *AT5G07220/AtBAG3*	*I. batatas*	*g1642.t1*	*IbBAG1*
		g20550.t1	*IbBAG5*
		g23383.t1	*IbBAG6*
		g25310.t1	*IbBAG7*
	I. trifida	*itf03g12810.t1*	*ItfBAG3*
		itf05g14330.t1	*ItfBAG6*
		itf12g25060.t1	*ItfBAG13*
	I. triloba	*itb03g13440.t1*	*ItbBAG3*
		itb05g14970.t1	*ItbBAG6*
		itb12g25410.t1	*ItbBAG13*
Group Ⅱ: *AT3G51780/AtBAG4* *AT1G12060/AtBAG5*	*I. batatas*	*g4176.t1*	*IbBAG2*
		g15882.t1	*IbBAG3*
		g34788.t1	*IbBAG10*
		g39432.t1	*IbBAG11*
		g46511.t1	*IbBAG12*
		g49380.t1	*IbBAG13*
		g49384.t1	*IbBAG14*
		g58853.t1	*IbBAG15*
	I. trifida	*itf01g03220.t1*	*ItfBAG1*
		itf03g01800.t1	*ItfBAG2*
		itf05g00940.t1	*ItfBAG5*
		itf07g14940.t1	*ItfBAG7*
		itf08g09020.t1	*ItfBAG8*
		itf09g09430.t1	*ItfBAG9*
		itf10g17930.t1	*ItfBAG10*
		itf13g03550.t1	*ItfBAG14*
	I. triloba	*itb01g03520.t1*	*ItbBAG1*
		itb03g01750.t1	*ItbBAG2*
		itb05g00360.t1	*ItbBAG5*
		itb07g16910.t1	*ItbBAG7*
		itb08g09640.t1	*ItbBAG8*
		itb09g10270.t1	*ItbBAG9*
		itb10g18190.t1	*ItbBAG10*
		itb13g05240.t1	*ItbBAG14*
Group Ⅲ: *AT2G46240/AtBAG6* *AT5G62390/AtBAG7*	*I. batatas*	*g20498.t1*	*IbBAG4*
		g25374.t1	*IbBAG8*
		g30328.t1	*IbBAG9*
	I. trifida	*itf03g13330.t1*	*ItfBAG4*
		itf11g00190.t1	*ItfBAG11*
		itf12g24570.t1	*ItfBAG12*

		itb03g13970.t1	*ItbBAG4*
	I. triloba	*itb11g00170.t1*	*ItbBAG11*
		itb12g24940.t1	*ItbBAG12*

Table S3. Primers used in this study.

Gene	Forward Primer	Reverse Primer
IbBAG1	GTGCAGAAACGGGGTCCA	CCCAGTTGGCCCTGTCAG
IbBAG4	AAGACGAGCTCGATGCCG	CGGTCACGGCATCCAAGT
IbBAG5	ACTCCACAGCAGCAGCAG	GTGGGCGTCGGAAAGTGA
IbBAG7	AACAACAGCCGGGGATGG	CTGCCCCGAGAGCTGAAG
IbBAG8	GGGTCTTGGCCCTGGAAC	CGCCGTCCACGTGTACTT
IbBAG9	CAAGGTCTGAAGGCGCCA	GGGGTTTTCTCCGCCGAA
IbBAG11	GAGGTTGTTTTCATGGCGGC	AGTGCTACCAACAATGGCAGA
IbBAG15	GGCGGTGAGGAAATCGCT	GGTAACAGTGGCGGCAGT

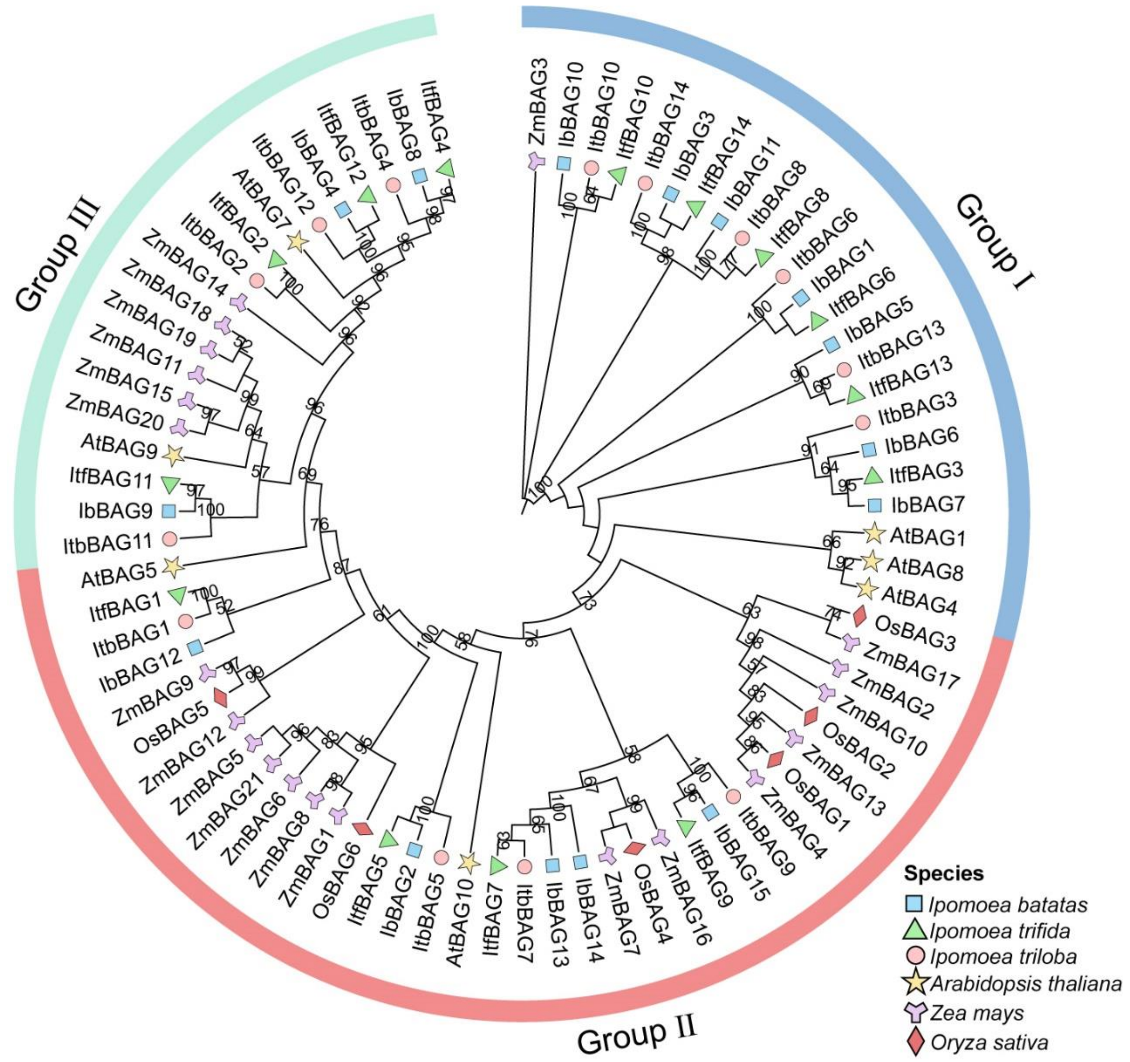

Figure S1. Phylogenetic analysis of the BAG proteins from seven plant species (i.e., *I. batatas*, *I. trifida*, *I. triloba*, *Arabidopsis thaliana*, *Oryza sativa* L., and *Zea mays*). A total of 77 BAGs were divided into three subgroups (Group Ⅰ to Group Ⅲ*)* according to the evolutionary distance. The pink circles, blue squares, green triangles, yellow stars, purple wyes and red diamonds respectively represent the 15 IbBAGs in *I. batatas*, 14 ItfBAGs in *I. trifida*, 14 ItbBAGs in I. *triloba*, 7 AtBAGs in *Arabidopsis thaliana,* 6 OsBAGs in *Oryza sativa* L., and 21 ZmBAGs in *Zea mays*.

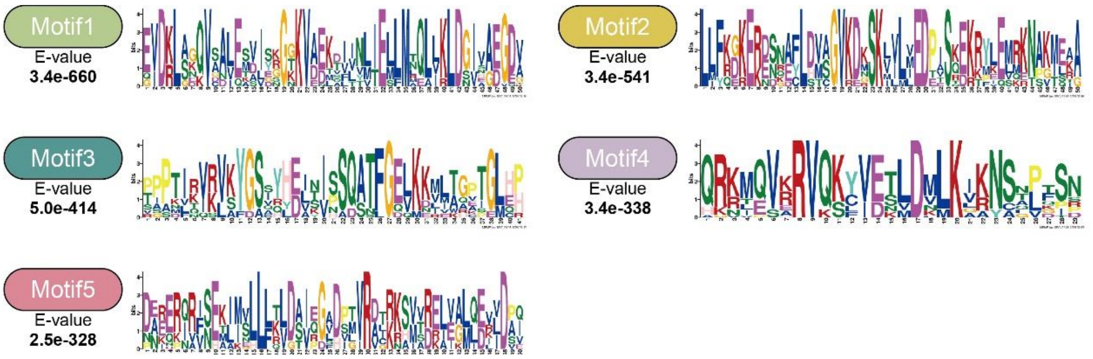

Figure S2. Conserved motifs analysis of IbBAGs in *I. batatas*.

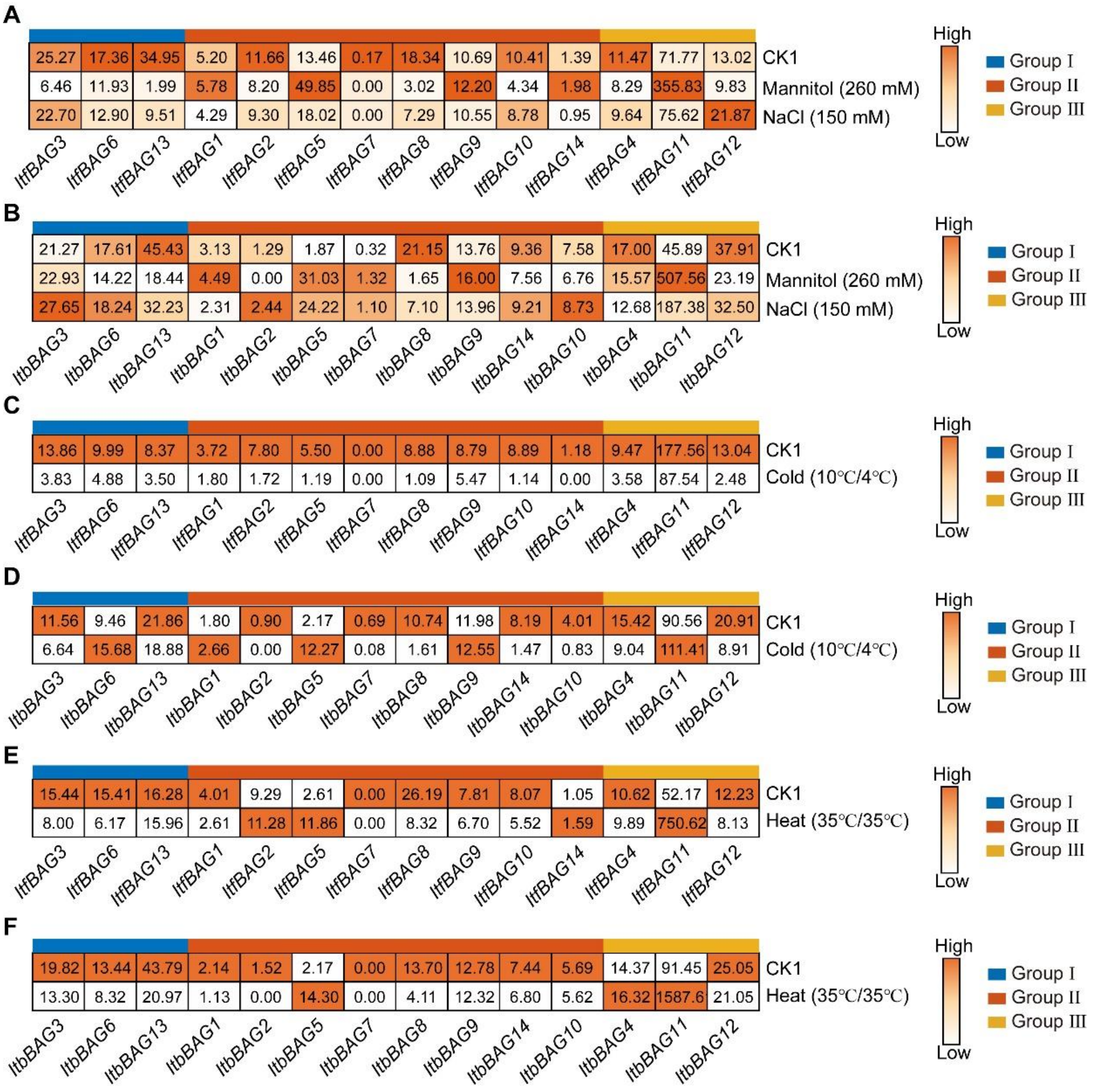

Figure S3. Gene expression patterns of *BAGs* in response to different abiotic stress (Mannitol, NaCl, cold and heat) in *I. trifida* and *I. triloba* as determined by RNA-seq. FPKM was shown in the boxes.
